# Effects of Revegetation on Soil Organic Carbon Storage and Erosion-Induced Carbon Loss under Extreme Rainstorms in the Hill and Gully Region of the Loess Plateau

**DOI:** 10.3390/ijerph13050456

**Published:** 2016-04-29

**Authors:** Yujin Li, Juying Jiao, Zhijie Wang, Binting Cao, Yanhong Wei, Shu Hu

**Affiliations:** 1 State Key Laboratory of Soil Erosion and Dry Land Farming on the Loess Plateau, Institute of Soil and Water Conservation, Northwest A&F University, No. 26, Xinong Road, Yangling 712100, China; lyjin1919@gmail.com (Y.L.); 15291868362@163.com (B.C.); hushumemory2008@126.com (S.H.); 2 Institute of Soil and Water Conservation, Chinese Academy of Sciences and Ministry of Water Resources, Yangling 712100, China; sdliyj2008@126.com (Z.W.); yhweigo@163.com (Y.W.)

**Keywords:** revegetation, plant community, soil erosion, carbon storage, carbon loss, Loess Plateau

## Abstract

*Background*: The Loess Plateau, an ecologically vulnerable region, has long been suffering from serious soil erosion. Revegetation has been implemented to control soil erosion and improve ecosystems in the Loess Plateau region through a series of ecological recovery programs. However, the increasing atmospheric CO_2_ as a result of human intervention is affecting the climate by global warming, resulting in the greater frequency and intensity of extreme weather events, such as storms that may weaken the effectiveness of revegetation and cause severe soil erosion. Most research to date has evaluated the effectiveness of revegetation on soil properties and soil erosion of different land use or vegetation types. Here, we study the effect of revegetation on soil organic carbon (SOC) storage and erosion-induced carbon loss related to different plant communities, particularly under extreme rainstorm events. *Materials and methods*: The erosion-pin method was used to quantify soil erosion, and soil samples were taken at soil depths of 0–5 cm, 5–10 cm and 10–20 cm to determine the SOC content for 13 typical hillside revegetation communities in the year of 2013, which had the highest rainfall with broad range, long duration and high intensity since 1945, in the Yanhe watershed. *Results and discussion*: The SOC concentrations of all plant communities increased with soil depth when compared with slope cropland, and significant increases (*p* < 0.05) were observed for most shrub and forest communities, particularly for natural ones. Taking the natural secondary forest community as reference (*i.e.*, soil loss and SOC loss were both 1.0), the relative soil loss and SOC loss of the other 12 plant communities in 2013 ranged from 1.5 to 9.4 and 0.30 to 1.73, respectively. Natural shrub and forest communities showed greater resistance to rainstorm erosion than grassland communities. The natural grassland communities with lower SOC content produced lower SOC loss even with higher soil loss, natural secondary forest communities produced higher SOC loss, primarily because of their higher SOC content, and the artificial *R. pseudoacacia* community with greater soil loss produced higher SOC loss. *Conclusions*: These results indicate that natural revegetation is more effective in enhancing SOC storage and reducing soil erosion than artificial vegetative recovery on hillsides. However, natural secondary forest communities, with higher SOC content and storage capacity, may also contribute to larger SOC loss under extreme rainstorms.

## 1. Introduction

Soil, the largest pool of terrestrial organic carbon in the biosphere and approximately 3.3 times the size of the atmospheric pool and 4.5 times the size of the biotic pool [1,2], plays an important role in the terrestrial carbon cycle. Soil carbon (C) storage is a strategy to achieve food security and mitigate climate change while simultaneously increasing productivity, improving water quality, and restoring degraded soils and ecosystems [2]. Soils act as a carbon sink to store carbon dioxide through afforestation and other land use conversions [3,4,5]. However, soil erosion has significant impacts on soil degradation and the global carbon cycle [6,7]. Lateral C-fluxes are primarily influenced by water erosion, which disturbs the soil surface and transports sediment-bound carbon out of the system [8]. Recent studies suggest that climatic variability will increase as a consequence of global warming and will result in a greater frequency and intensity of extreme weather events, such as storms, leading to a decrease in regional ecosystem carbon stocks, which has the potential to negate a predicted increase in terrestrial carbon uptake [9,10]. As an essential element of soil physical and chemical properties, soil organic carbon (SOC) plays an important role in enhancing crop production and mitigating greenhouse gas emissions [11,12]. Strategies for restorative land use and the adoption of recommended management practices on cropland, grazing lands and forestlands can restore the SOC pool while mitigating climate change [13]. Therefore, it is important for regional ecosystem sustainability to study the effects of land cover conversion on soil carbon storage and erosion-induced carbon loss, particularly under extreme rainstorm events.

The Chinese Loess Plateau has long experienced serious soil erosion, and severe soil and water loss has led to widespread land degradation [14]. Land use and surface cover are the principal determinants of erosion rates [15]. Unsustainable land use and low vegetative cover are the primary reasons for soil erosion and nutrient loss in the Loess Plateau [16,17,18,19]. To implement ecological restoration and soil erosion control, a series of revegetation measures have been conducted since the 1950s in the Loess Plateau, in particular the “Grain for Green” project (GFG) in 1999. This project promoted the conversion of slope arable land to forest or grassland. Revegetation enhanced the storage of SOC in soils by reducing soil erosion and increasing inputs of organic materials [20]. Anthropogenic changes in vegetative cover and natural succession have been shown to be a significant ways of carbon sink [21]. However, on-site SOC losses caused by water erosion under different land uses have been found to have significant effects on lateral carbon fluxes and deserve attention in the context of the global carbon cycle [8,22,23].

Conversion of land use to agriculture has many potential implications for the mobilization and export of SOC [24]. Flooding events in agricultural watersheds are believed to be particularly important because they mobilize large quantities of total organic carbon (TOC) into the stream channel [25,26]. However, land use and land cover (LULC) have a strong influence on the intensity of soil erosion processes and the consequent loss of SOC and nutrients from soils. Among forest, shrubland, pasture and agriculture classes in a Mediterranean catchment, agricultural areas were found to be the most dynamic sites, accounting for 70% of the eroded soil but 45% of the total eroded C, and the remaining percentage of eroded C was derived primarily from relatively C-rich forest soils, which were the dominant LULC class in terms of spatial extent [23]. 

Revegetation probably enhances the carbon storage capacity of terrestrial ecosystems on the Loess Plateau [27,28]. The different vegetation characteristics and organic carbon content in soils should influence the extent of soil erosion and its associated carbon loss within different plant communities. Following the implementation of a series of vegetation recovery programs in the Loess Plateau region, vegetation community coverage, height, diversity and stability increased as a whole, and thus, vegetation quality and quantity significantly increased accordingly [29]. Tree planting was widely implemented to accelerate community succession because the process of natural succession was very slow in the Loess Plateau region. In addition, planting trees and grasses could increase surface vegetation cover and control soil erosion under certain conditions [17,19,30]. Successful models of afforestation were also found for “ecological forests” (*i.e.*, trees for soil and water conservation, such as *Robinia pseudoacacia*, *Pinus tabuliformis*, *Caragana intermedia*, and *Hippophae rhamnoides*) and “economic forests” (*i.e.*, horticultural tree crops, such as *Malus pumila* and *Armeniaca vulgaris*) [29]. However, low production “small but old tree” forests and tree death in drought years also existed widely, depending on the long-term soil water deficit, soil desiccation and the decrease of streamflow in different scale catchments [31,32,33,34]. Natural vegetation on abandoned land may begin the process of succession after the termination of human disturbance and gradually transform to secondary wild grassland or secondary forest communities [35,36]. In the hill and gully landscape of the Loess Plateau, inter-gullies are the primary location of croplands, and the abandonment of cropland is related to slope gradient, distance from the village, and soil and water conservation policies; as a result, plant communities with different successional stages have developed, such as communities dominated by *Artemisia scoparia*, *Lespedeza davurica*, *Artemisia gmelinii*, *Bothriochloa ischaemun*, *Sophora viciifolia*, and *Quercus liaotungensis*, respectively [37,38]. Although the implementation of a series of ecological recovery programs has achieved remarkable progress in soil erosion control [39,40], it is still likely that soil loss will be extremely severe during heavy rainstorms [41]. Owing to the differential ability of vegetation types to reduce soil loss under extreme rainstorm events [42], corresponding differences in carbon losses also exist. Currently, most related studies in the Loess Plateau region have focused on soil erosion and soil organic carbon distribution under different land uses or vegetation types at the slope scale [43,44] or the watershed scale [45,46,47]. However, little is known about SOC storage and erosion-induced SOC loss related to differences among plant communities, particularly erosion-induced SOC loss under extreme rainstorm events. It happened that Yanhe watershed experienced consecutive heavy rainfalls with broad range, long duration and high intensity in July 2013.

Thus, the objectives of this study were to examine the effects of revegetation on soil organic carbon storage and carbon losses caused by soil erosion on hillsides in response to extreme rainstorm events in the hill and gully area of the Loess Plateau by analyzing: (1) changes in soil organic carbon content and capacity for soil carbon storage; and (2) soil erosion and erosion-induced carbon loss of different plant communities. The results of this study could provide references for strategies towards the optimization of vegetation restoration and climate change mitigation in the Loess Plateau region.

## 2. Materials and Methods

### 2.1. Study Area

**Figure 1:**
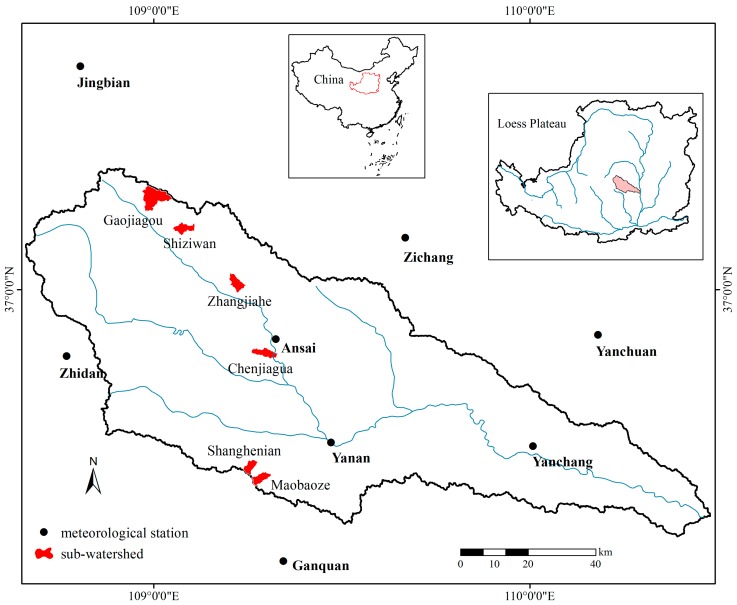
Location and distribution of the six sub-watersheds and meteorological stations in the Yanhe watershed.

The study area was located in the Yanhe watershed at N 36°23′–37°17′ and E 108°45′–110°28′ (Figure 1). The watershed that occupies 7687 km^2^ is the primary tributary in the middle reaches of the Yellow River and belongs to the hill-gully region of the Loess Plateau. The climate is semi-arid, with mean annual precipitation of 500 mm (420–540 mm), and mean air temperature of 9.5 °C (8.80 °C to 10.2 °C). The seasonal distribution of precipitation is uneven, characterized by intensive rainfall from July to September that accounts for more than 60% of the annual rainfall. Soils are predominantly loess and have a texture of 64%–73% silt and 17%–20% clay; the loose soils are vulnerable to dispersion and transport [48,49]. 

The natural vegetation is composed of grassland, shrub and secondary forest. The dominant tree species include *Quercus liaotungensis*, *Pinus tabuliformis*, *Platycladus orientalis*, and *Acer buergerianum.* The dominant shrub species include *Syringa julianae*, *Sophora viciifolia*, *Periploca sepium*, and *Ostryopsis davidiana*. And the dominant herb species include *Artemisia scoparia*, *Stipa grandis*, *Stipa bungeana*, *Artemisia gmelinii*, *Artemisia giralaii*, *Thymus mandshuricus*, *Bothriochloa ischaemum*, *Lespedeza davurica*, and *Carex lanceolata*. The primary introduced tree species include *Robinia pseudoacacia, Populus simonii*, *Hippophae rhamnoides*, *Caragana intermedia*, and *Salix matsudana*. The economic forests are dominated by *Malus pumila*, *Prunus davidiana*, and *Armeniaca sibirica* [29]*.*

### 2.2. Experimental Design and Soil Sampling

Our research was conducted in six sub-watersheds distributed from south to north in the Yanhe watershed (Figure 1). The survey sites were in inter-gully areas and 13 communities, defined by species dominance, were selected. There were four grassland communities of *Artemisia scoparia* (A), *Stipa bungeana + Lespedeza davurica* (SL), *Artemisia gmelinii + Stipa bungeana* (AS) and *Artemisia gmelinii + Artemisia giralaii+Bothriochloa ischaemum*(AAB); two natural shrubland communities of *Sophora viciifolia* − *Artemisia gmelinii + Stipa bungeana* (SAS) and *Syringa julianae* − *Carex lanceolata* (SC); two natural forestland communities of *Quercus liaotungensis* − *Syringa julianae* − *Carex lanceolata* (QSC) and *Acer buergerianum* − *Syringa julianae* − *Carex lanceolata* (ASC), two artificial shrubland communities of *Hippophae rhamnoides* − *Artemisia gmelinii + Stipa bungeana* (HAS) and *Caragana intermedia* − *Artemisia gmelinii + Stipa bungeana* (CAS); and three artificial forest communities of *Armeniaca sibirica* − *Artemisia gmelinii + Stipa bungeana* (AAS), 6–8 years *Robinia pseudoacacia* − *Artemisia gmelinii + Stipa bungeana* (RAS1) and 15 years *Robinia pseudoacacia* − *Artemisia gmelinii + Stipa bungeana* (RAS2). Each community had three replicate sites distributed in different sub-watersheds, and three replicates of slope croplands (*P. miliaceum*, *Z. mays*) were used as the reference (Table 1).

The erosion-pin method [50] was used to set up observation plots in May 2013 (before the rainy season). Three 2 m × 2 m plots, each with nine pins at intervals of 50 cm, were laid out at each site in the 13 plant communities. The erosion pins were iron nails 10 cm in length and 5 mm in diameter. The pins were inserted with uniform hammering to minimize soil disturbance. The top of each nail was even with the soil surface. A red stick was inserted 5 cm to the right side of each pin to facilitate locating the pin. Meanwhile, vegetation, litter and biocrust cover of each site were recorded. The cover of the vegetation in plots was estimated visually by two or three observers working together. In late October 2013, the erosion depth (−) or deposition depth (+) of the pins was measured to the nearest 0.1 mm using digital caliper. Fortunately, the largest rainstorms happened in July 2013 since 1945, and the rain was extraordinarily large to differentiate soil erosion among these treatments.

At each site, soil samples were collected with a soil auger (internal diameter 4.8 cm) from nine points in an S-shaped pattern that paralleled the slope at depths of 0–5 cm, 5–10 cm and 10–20 cm. The soil samples from the nine points in each depth were mixed, respectively. Live plant materials (roots and shoots) and pebbles from each sample were separated in the laboratory and discarded. All samples were air-dried, crushed, and passed through a 0.15 mm sieve prior to the SOC analysis. SOC was determined by using the oil bath-K_2_CrO_7_ titration method (the Agricultural Chemistry Committee of Chinese Soil Academy, 1984). Soil bulk density of each plot (3 replicates) was measured collecting soil with a bulk sampler (100 cm^3^) near the soil sampling points, and comparing the original mass of each soil core with the mass after oven-drying at 105 °C for 24 h. 

### 2.3. Data Analysis

As the topsoil was disturbed and transported once water erosion had taken place, the organic carbon percentage content (%) of 0–5 cm soil layer was used as the base value to calculate soil organic carbon loss. The soil erosion and soil organic carbon loss in different plant communities were calculated using the following equations:

(1) $$X_{j}=\left(\sum_{i=1}^{m}D_{i}\times B_{i}\times 10^{3}\right)/m\;\left(m=3\right)$$

(2) $$S=\left(\sum_{j=1}^{n}X_{j}\right)/n\;\left(n=3\right)$$

(3) $$SL=\left(\sum_{j=1}^{n}X_{j}\times P_{j}\right)/n\;\left(n=3\right)$$

where *X_j_* is the average soil erosion of each site, *D_i_* is the average depth (mm) of the nine nails in each plot, *B_i_* is the average soil bulk density (g·cm^−3^) of each plot, 10^3^ is the unit conversion factor, *S* is the soil erosion (t·km^−2^·yr^−1^) of each plant community, *SL* is the soil organic carbon loss (t·km^−2^·yr^−1^) of each plant community, and *P_j_* is the average soil surface (0–5 cm) organic carbon percent content (%) of each site; *i* is the plot number of each site and m is the total number of plots, *j* is the site number of each plant community and n is the total number of sites.

As the absolute values were obtained in a year experiencing extraordinary storm events, relative soil loss and soil organic carbon loss values were considered to be more meaningful. Therefore, the natural forestland community *Q. liaotungensis* − *S. julianae* − *C. lanceolata* (QSC), with the lowest soil erosion and the highest soil organic carbon, was used as a reference to calculate relative soil loss and soil organic carbon loss of the other communities. The relative soil loss and soil organic carbon loss in different plant communities were calculated using the following equations:

*R_S_* = *S_k_*/*S_QSC_* (4)

*R_SL_* = *SL_k_*/*SL_QSC_* (5)

where *R_S_* and *R_SL_* is the relative soil loss and relative soil organic carbon loss, respectively, *S_k_* and *SL_k_* is the soil erosion and soil organic carbon loss in different plant communities, *k* represents the 13 plant communities, *S_QSC_* and *SL_QSC_* is the soil erosion and soil organic carbon loss of the natural forestland community *Q. liaotungensis* − *S. julianae* − *C. lanceolata* (QSC), respectively.

A two-way analysis of variance (ANOVA) was used to examine the effects of plant community and soil depth on soil organic carbon content. A one-way ANOVA was used to identify the effects of plant community on relative soil loss and relative soil organic carbon loss. If significant effects were detected with the ANOVA, differences between individual means were tested using the Tukey Honestly Significant Difference (HSD) test at *p* < 0.05. Statistics were run using SPSS 16.0 (SPSS Inc., Chicago, IL, USA). Figures were drawn using Sigmaplot 12.0 (Systat Software Inc., San Jose, CA, USA).

## 3. Results

### 3.1. Changes in Soil Organic Carbon Content 

The two-way ANOVA indicated that plant community and soil depth had significant effects on SOC content (Table 2). Compared with the slope cropland, the SOC content of the 13 plant communities was increased or significantly increased (*p* < 0.05) at all soil depths. The SOC content in each soil layer of the natural secondary forest communities was significantly higher (*p* < 0.05) than in other natural plant communities. In addition, the SOC content of the AS community was significantly higher (*p* < 0.05) at each soil depth than in other natural grassland communities, and there was no significant difference between the other natural grassland communities. The SOC content of the natural secondary forest and shrub communities was significantly higher (*p* < 0.05) than the SOC content of artificial forest and shrub communities, respectively. In addition, the SOC content of each soil layer was not significantly different between artificial forest communities (except for the RAS1) or between artificial shrub communities. 

The SOC content of the different plant communities ranged from 4.86 to 52.89 g·kg^−1^ at 0–5 cm, from 3.89 to 26.71 g·kg^−1^ at 5–10 cm, and from 2.71 to 18.44 g·kg^−1^ at 10–20 cm (Table 2). As soil depth increased, the soil organic carbon concentration decreased (*p* < 0.05), but this change was not significant for the grassland communities. SOC content was significantly higher (*p* < 0.05) in the 0–5 cm soil layer than in the deeper soil layers of the forest community (except for RAS1) and shrub communities (except for the HAS). 

### 3.2. Extreme Rainfall and Soil Erosion 

In July 2013, the Yanhe watershed experienced consecutive heavy rainfalls with broad range, long duration and high intensity. At each meteorological station (Figure 1), the total quantity of rainfall in July ranged from 327 to 793 mm, the frequency of rainstorms ranged from two to eight storms, and this series of storms accounted for 65%–100% of total rainstorms amount (126–678 mm) for the year. The Yan’an station received the highest rainfall in July, which exceeded the average annual rainfall (500 mm). This was the highest rainfall since 1945 in this watershed. The total annual precipitation at each meteorological station ranged from 612 mm to 1357 mm, primarily concentrated between June and September, and represented 92%–96% of the total annual precipitation (Table 3). 

**Figure 2:**
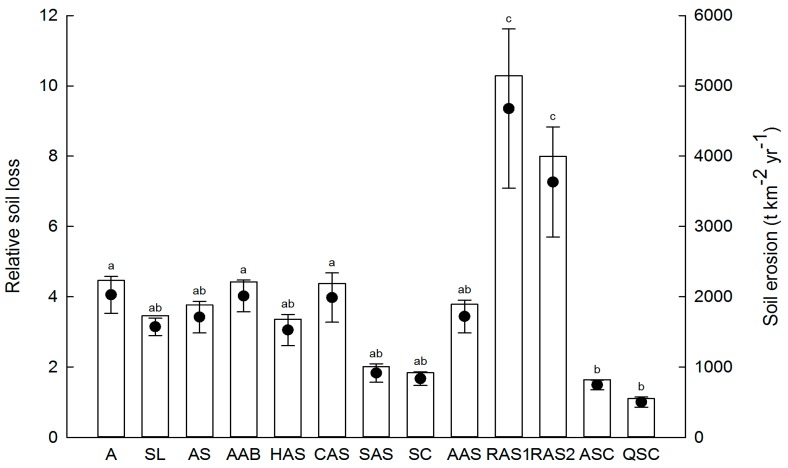
Soil erosion and relative soil loss in different plant communities under the extreme rainfall events during 2013. Vertical columns and black circles represent soil erosion and relative soil loss, respectively. Different lowercase letters indicate significance at *p* < 0.05 for relative soil loss between different plant communities. A: *Artemisia scoparia* (3–5 years); SL: *S. bungeana + L. davurica* (10–20 years); AS: *A. gmelinii + S. bungeana* (25–30 years); AAB: *A. gmelinii + A. giralaii + B. ischaemum* (30 years); HAS: *H. rhamnoides − A. gmelinii + S. bungeana* (15–20 years); CAS: *C. intermedia − A. gmelinii + S. bungeana* (15–20 years); SAS: *S. viciifolia − A. gmelinii + S. bungeana* (30–40 years); SC: *S. julianae − C. lanceolata* (30–40 years); AAS: *A. sibirica − A. gmelinii + S. bungeana* (15 years); RAS1: *R. pseudoacacia − A. gmelinii + S. bungeana* (6–8 years); RAS2: *R. pseudoacacia − A. gmelinii + S. bungeana* (15 years); ASC: *A. buergerianum − S. julianae − C. lanceolata* (40–50 years); QSC: *Q. liaotungensis − S. julianae − C. lanceolata* (60–70 years).

Under the extreme rainfall events of 2013, the soil loss of the 13 communities ranged from 550 t·km^−2^·yr^−1^ to 5145 t·km^−2^·yr^−1^. The QSC community had the lowest soil loss and was used as a reference to calculate the relative soil loss of the other plant communities. The relative soil loss within the four natural grassland communities ranged from 3.1 to 4.1, and the A community had the lowest resistance to erosion. There was no significant difference in relative soil loss between the two natural shrub communities, which showed a higher resistance to erosion than the grassland communities. Relative soil loss of the secondary forest communities was 1 and 1.5 and showed the greatest efficacy for reducing erosion. In the two artificial shrub communities, relative soil loss was 3 and 3.9. Relative soil loss of the artificial forest communities ranged from 3.4 to 9.4, and the 6–8 years *R. pseudoacacia* community showed the lowest resistance to erosion among the 13 plant communities (Figure 2).

### 3.3. Soil Organic Carbon Loss 

**Figure 3:**
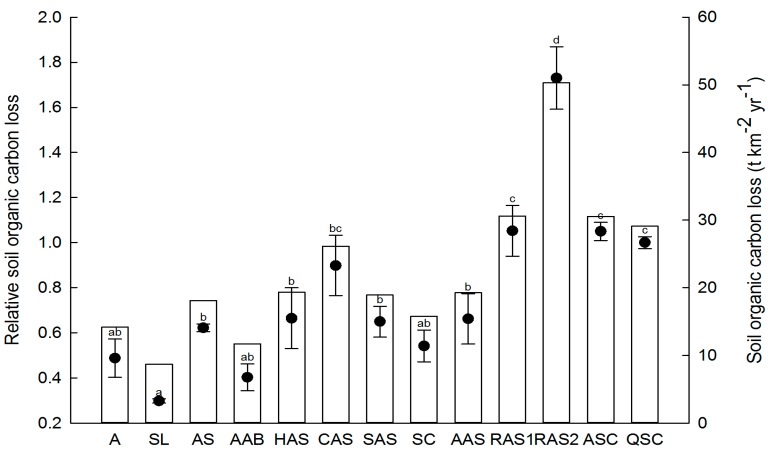
Soil organic carbon loss and relative soil organic carbon loss in different plant communities under extreme rainstorm events during 2013. Vertical columns and black circles represent soil organic carbon loss and relative soil organic carbon loss, respectively. Different lowercase letters indicate significance at *p* < 0.05 for relative soil organic carbon loss between different plant communities. A: *Artemisia scoparia* (3–5 years); SL: *S. bungeana + L. davurica* (10–20 years); AS: *A. gmelinii + S. bungeana* (25–30 years); AAB: *A. gmelinii + A. giralaii + B. ischaemum* (30 years); HAS: *H. rhamnoides − A. gmelinii + S. bungeana* (15–20 years); CAS: *C. intermedia − A. gmelinii + S. bungeana* (15–20 years); SAS: *S. viciifolia − A. gmelinii + S. bungeana* (30–40 years); SC: *S. julianae − C. lanceolata* (30–40 years); AAS: *A. sibirica − A. gmelinii + S. bungeana* (15 years); RAS1: *R. pseudoacacia − A. gmelinii + S. bungeana* (6–8 years); RAS2: *R. pseudoacacia − A. gmelinii + S. bungeana* (15 years); ASC: *A. buergerianum − S. julianae − C. lanceolata* (40–50 years); QSC: *Q. liaotungensis − S. julianae − C. lanceolata* (60–70 years).

In 2013, with extreme rainstorms, the soil organic carbon loss of the 13 communities ranged from 8.67 t·km^−2^·yr^−1^ to 50.34 t·km^−2^·yr^−1^. The QSC community experienced the lowest soil erosion and had the highest soil organic carbon content, and was also used as a reference to calculate the relative soil organic carbon loss of the other communities. The relative soil organic carbon loss ranged from 0.30 to 0.62 for the grassland communities, the AS community showed the greatest loss, and the SL community showed the least loss. No significant difference was found between natural and artificial shrub communities, and the relative SOC loss ranged from 0.54 to 0.89. In artificial forest communities, significant differences were found between the *R. pseudoacacia* communities (1.73 for 15 years and 1.05 for 6–8 years) and the AAS community (0.66). No significant difference was found between the two natural forest communities (1.05 and 1). The 15 years *R. pseudoacacia* community had the highest relative SOC loss between the 13 plant communities (Figure 3).

## 4. Discussion

### 4.1. Soil Organic Carbon Sequestration by Vegetation

Land use changes have significant effects on SOC concentrations and the carbon cycle of terrestrial ecosystems. Total SOC concentrations and stocks decreased significantly after grassland was converted to cropland in a semi-arid grassland [51]. In northwestern Spain, Mireia *et al.* [52] reported approximately 67% of the total carbon loss in the topsoil was a result of the conversion of natural *Quercus ilex* forest into cropland, and after reforestation with *Pinus halepensis*, the soil organic carbon increased significantly. In our study, the soil organic carbon (SOC) content of all the vegetation types were higher than slope cropland (Table 2). This corresponds to the results of most studies, which have demonstrated that revegetation increases the soil organic carbon concentrations [5,27,53]. However, differences in SOC content were also found among vegetation types, the rank of average SOC content at each soil depth was as follows: natural secondary forestland > natural shrubland > artificial shrubland > artificial forestland > natural grassland > slope cropland. This order implies that the SOC content was improved through vegetative recovery and that natural secondary forestland had the greatest capacity to store soil organic carbon, particularly in the 0–5 cm soil layer. Compared with slope cropland, the SOC concentration in the surface soil (0–5 cm) increased by 3.29%–98.15%, 253.50%–286.63% and 666.46%–988.27% when it was converted to natural grassland, natural shrubland and natural secondary forestland, respectively. Vegetative cover has fundamental effects on soil properties [54], the lower plant and litter cover of natural grassland communities would have limited the contribution of organic matter to the soil (Table 1). The dense canopy structure and species composition of natural shrubland and forestland, with a higher ability to increase the amount of organic matter in the soil and reduce SOC decomposition rates than natural grassland, have been found to have a positive effect on SOC accumulation [55].

With the conversion of slope cropland to artificial shrubland and forestland, the SOC content of the soil profile (0–20 cm) also showed increased tendency (Table 2). Similar results were found by Wang *et al.* [21], who showed that the surface (0–20 cm) SOC content increased over eight years of revegetation when cultivated crops were converted to grass (primarily *Artemisia Linn*, couch grass and small weeds), artificial shrubs (*Prunus armniaca*) and immature forest plantations (*Robinia pseudoacacia*). In our study, compared with slope cropland, the SOC content for *R. pseudoacacia* land was not significantly higher after six to eight years of revegetation, but a significant increase was observed at the soil surface (0–5 cm) after 15 years (Table 2). Lu *et al.* [56] found that the time required for SOC to transition from source to sink in the Loess Plateau region was three to eight years of afforestation. Qin *et al.* [57] found that soil organic carbon density (SOCD) in the 0–20 cm soil layer of an immature woodland (<10 a) was lower than that of cultivated land, but the SOCD of mature woodland (>30 a) was 59.54% higher than that of cultivated land. These studies and our data suggest that the time since afforestation should be considered when evaluating the SOC sequestration capacity. 

### 4.2. Soil Organic Carbon Loss through Soil Erosion 

With the implementation of “Green for Green” project (GFG), large areas of slope cropland were converted to trees, shrubs, or grasses within the Loess Plateau region [32,58]. Plant structure and cover were the keys to reducing runoff and soil loss by improving water infiltration, reducing the impact of water droplets, intercepting rain and snow and physically stabilizing the soil with roots and leaf litter [59,60]. In the grasslands, biocrust covered most of the soil surface, which played a more important role in soil erosion control than the vascular plant canopy in the hilly Loess Plateau [61]. Pickup *et al.* [62] found that sediment moves as a series of scour-transport-fill sequences in the arid zone. In our study, soil erosion and deposition coexisted on the hillside, but erosion dominated in all the plant communities in the year of 2013 with extreme rainstorm events in the Yanhe watershed. 

Under extreme rainstorm events in 2013, grass communities showed a lower resistance to soil erosion (Figure 2), which may be attributed to the uneven distribution of biocrust and plants on the one hand and the low resilience to physical disturbance and increasing human activity on the other hand; similar results were found in another arid zone by Warren [63]. Shrub communities (except for the CAS), which had a lower soil erosion than grassland communities, were better in protecting the soil (Figure 2). Wei *et al.* [42] reported that *H. rhamnoides*, with a mean canopy cover of 92% and a litter depth 30 mm, is a powerful agent of erosion control during extreme hydrological events (maximum 30 min rainfall intensity >0.55 mm/min). However, *H. rhamnoides* did not achieve a similar effectiveness in controlling soil erosion in our study, which can be accounted for by the significantly lower canopy cover and litter depth (40% and 1.5 mm, respectively). Natural secondary forest communities, especially the QSC, were most effective at protecting the soil from erosion, which can be unequivocally attributed to the dense canopy structure and the higher litter cover (Table 1). Similar results in southeastern Spain suggest that forests with canopies of 60%–85% can effectively reduce rainfall erosivity [59]. It has been shown that afforestation results in soil desiccation and has no additional advantages in terms of species diversity and soil erosion resistance over natural recovery [27]. In our study, the 6–8 years *R. pseudoacacia* community, with lower tree cover, grass cover and litter cover, had the highest soil erosion (Table 1; Figure 2). This is consistent with the result that young woodland (1–6 years) showed higher soil erosion than ripewood (6–15 years) [48].

The soil carbon pool is tightly linked to soil erosion, and soil erosion can result in net carbon loss from the carbon pool [6,8,22]. However, revegetation reduces soil erosion and enhances soil organic carbon storage capacity [21,64]. In our study, natural secondary forest communities showed higher SOC content, and artificial *R. pseudoacacia* communities showed higher relative soil loss and carbon loss. More carbon-concentrated plant communities may also produce greater SOC loss when water erosion takes place at the soil surface during extreme rainstorm events, as was found by Nadeu *et al.* [23] in a Mediterranean catchment. Artificial forests have poor cover, limited understory and compacted soil due to water shortages in the loess hilly area [5], and soil erosion cannot be effectively controlled. Thus, extreme rainfall events may currently play a key role in soil organic carbon loss in the hill and gully areas of the Loess Plateau.

## 5. Conclusions

These results might have implications for vegetation restoration projects with respect to revegetation pathway selection and global climate change mitigation. We can conclude from the present study that vegetation restoration can increase or significantly increase the SOC content of surface soil (0–20 cm), especially in the 0–5 cm soil layer, and natural vegetative recovery was a more effective pathway for increasing SOC concentration and enhancing SOC storage than artificial vegetative recovery in the Chinese loess hill and gully region. 

Soil erosion and erosion-induced carbon loss is tightly linked with vegetation types under extreme rainstorm events. Grassland communities, with a higher soil loss and a lower SOC content, had less SOC loss; shrub communities were intermediate in SOC content and loss; artificial *R. pseudoacacia* communities, with higher soil loss, produced greater SOC loss; and natural secondary forest communities, with higher carbon-concentrated soils and SOC storage capacity, may produce more SOC loss from water erosion under extreme rainstorm events.

## Figures and Tables

**Table 1:** Sites conditions and the cover of different layers of plant communities.

Vegetation Type	Plant Community ^a^	Aspect ^b^	Slope (°)	Tc ^c^ (%)	Sc ^d^ (%)	Gc ^e^ (%)	Tpc ^f^ (%)	Lc ^g^ (%)	Bc ^h^ (%)
Slope cropland	P	SW	24 ± 2						
Natural grassland	A	SW	23 ± 3			26 ± 11	26 ± 11	19 ± 12	40 ± 16
	SL	SW	22 ± 4			15 ± 10	15 ± 10	16 ± 10	44 ± 16
	AS	SW	24 ± 1			42 ± 13	42 ± 13	24 ± 10	34 ± 14
	AAB	SW	25 ± 4			28 ± 13	28 ± 13	21 ± 13	44 ± 14
Artificial shrubland	HAS	NW	24 ± 4		41 ± 16	25 ± 9	67 ± 17	29 ± 8	32 ± 15
	CAS	SW	28 ± 1		41 ± 13	37 ± 17	78 ± 15	24 ± 9	19 ± 9
Natural shrubland	SAS	SW	29 ± 1		42 ± 5	23 ± 12	55 ± 17	25 ± 7	28 ± 9
	SC	NW	28 ± 2		32 ± 2	12 ± 3	44 ± 6	48 ± 17	33 ± 2
Artificial forestland	AAS	NW	27 ± 1	34 ± 19		17 ± 8	51 ± 14	20 ± 8	29 ± 7
	RAS1	NW	26 ± 2	9 ± 3		18 ± 6	27 ± 7	21 ± 11	22 ± 10
	RAS2	NW	25 ± 3	28 ± 8		25 ± 10	53 ± 12	31 ± 16	23 ± 13
Natural forestland	ASC	NW	27 ± 2	45 ± 7	25 ± 11	15 ± 9	85 ± 7	57 ± 5	5 ± 1
	QSC	NW	26 ± 2	60 ± 9	35 ± 6	10 ± 2	105 ± 6	87 ± 7	5 ± 2

**^a^** P: slope cropland cultivated with *P. miliaceum* or *Z. mays* (20 years); A: *Artemisia scoparia* (3–5 years); SL: *S. bungeana* + *L. davurica* (10–20 years); AS: *A. gmelinii* + *S. bungeana* (25–30 years); AAB: *A. gmelinii* + *A. giralaii* + *B. ischaemum* (30 years); HAS: *H. rhamnoides* − *A. gmelinii* + *S. bungeana* (15–20 years); CAS: *C. intermedia* − *A. gmelinii* + *S. bungeana* (15–20 years); SAS: *S. viciifolia* − *A. gmelinii* + *S. bungeana* (30–40 years); SC: *S. julianae* − *C. lanceolata* (30–40 years); AAS: *A. sibirica* − *A. gmelinii* + *S. bungeana* (15 years); RAS1: *R. pseudoacacia* − *A. gmelinii* + *S. bungeana* (6–8 years); RAS2: *R. pseudoacacia* − *A. gmelinii* + *S. bungeana* (15 years); ASC: *A. buergerianum* − *S. julianae* − *C. lanceolata* (40–50 years); QSC: *Q. liaotungensis* − *S. julianae* − *C. lanceolata* (60–70 years). **^b^** SW: 225°–270°; NW: 270°–315°. **^c^** Tree cover. **^d^** Shrub cover. **^e^** Grass cover. **^f^** Total plant cover (sum of tree cover, shrub cover and grass cover). **^g^** Litter cover. **^h^** Biocrust cover.

**Table 2:** Average values and standard deviations of the SOC content of different soil layers under different plant communities.

Vegetation Type	Plant Community ^e^	SOC (g·kg^−1^) ^f^		
		0–5 cm	5–10 cm	10–20 cm
Slope cropland	P	4.86 ± 0.98 ^a,A^	4.34 ± 0.71 ^a,A^	2.71 ± 0.52 ^a,A^
Natural grassland	A	6.36 ± 1.11 ^a,A^	5.54 ± 1.22 ^a,A^	3.78 ± 0.68 ^a,A^
	SL	5.02 ± 0.16 ^a,A^	3.89 ± 0.67 ^a,A^	3.51 ± 0.89 ^a,A^
	AS	9.63 ± 0.26 ^b,A^	8.20 ± 0.33 ^b,A^	6.28 ± 0.73 ^b,A^
	AAB	5.30 ± 0.78 ^a,A^	4.27 ± 0.95 ^a,A^	3.79 ± 0.97 ^a,A^
Artificial shrubland	HAS	11.52 ± 2.34 ^b,A^	8.14 ± 2.01 ^b,A^	5.65 ± 1.02 ^a,b,B^
	CAS	11.96 ± 1.79 ^b,A^	7.05 ± 0.58 ^b,B^	6.25 ± 0.33 ^b,B^
Natural shrubland	SAS	18.79 ± 1.98 ^c,A^	11.61 ± 1.21 ^c,B^	8.55 ± 1.52 ^c,C^
	SC	17.18 ± 2.23 ^c,A^	12.52 ± 3.28 ^c,B^	8.45 ± 1.72 ^c,C^
Artificial forestland	AAS	10.19 ± 1.72 ^b,A^	5.75 ± 0.60 ^a,,bB^	5.44 ± 0.68 ^a,b,B^
	RAS1	5.95 ± 0.64 ^a,A^	4.49 ± 0.49 ^a,A^	4.01 ± 0.42 ^a,b,A^
	RAS2	12.60 ± 1.01 ^b,A^	6.05 ± 0.98 ^a,b,B^	4.64 ± 0.64 ^a,b,B^
Natural forestland	ASC	37.25 ± 1.45 ^d,A^	24.28 ± 1.78 ^d,B^	15.34 ± 1.26 ^d,C^
	QSC	52.89 ± 1.39 ^d,A^	26.71 ± 4.71 ^d,B^	18.44 ± 3.07 ^d,C^

^A,B,C^ Different letters in the superscript within columns indicate significant different values according to HSD test at *p* < 0.05. ^a,b,c,d^ Different letters in the superscript within rows indicate significant different values according to HSD test at *p* < 0.05. **^e^** P: slope cropland cultivated with *P. miliaceum* or *Z. mays* (20 years); A: *Artemisia scoparia* (3–5 years); SL: *S. bungeana + L. davurica* (10–20 years); AS: *A. gmelinii + S. bungeana* (25–30 years); AAB: *A. gmelinii + A. giralaii + B. ischaemum* (30 years); HAS: *H. rhamnoides − A. gmelinii + S. bungeana* (15–20 years); CAS: *C. intermedia − A. gmelinii + S. bungeana* (15–20 years); SAS: *S. viciifolia − A. gmelinii + S. bungeana* (30–40 years); SC: *S. julianae − C. lanceolata* (30–40 years); AAS: *A. sibirica − A. gmelinii + S. bungeana* (15 years); RAS1: *R. pseudoacacia − A. gmelinii + S. bungeana* (6–8 years); RAS2: *R. pseudoacacia − A. gmelinii + S. bungeana* (15 years); ASC: *A. buergerianum − S. julianae − C. lanceolata* (40–50 years); QSC: *Q. liaotungensis − S. julianae − C. lanceolata* (60–70 years). **^f^** Soil organic carbon.

**Table 3:** Rainfall characteristics of the eight meteorological stations from May to October of 2013 in the Yanhe Watershed.

Month	Yanan		Ansai		Yanchang		Jingbian		Zhidan		Zichang		Yanchuan		Ganquan	
	Tr ^a^	Rr ^b^	Tr	Rr	Tr	Rr	Tr	Rr	Tr	Rr	Tr	Rr	Tr	Rr	Tr	Rr
May	29		26		3		8		40		19		20		52	
June	141	51 (1) ^c^	150		65		140		156		127		89		109	50 (1)
July	793	568 (8)	524	232 (3)	327	213 (3)	488	231 (4)	545	212 (3)	436	126 (2)	666	383 (5)	488	231 (3)
August	262	59 (1)	205	120 (2)	98		213	54 (2)	218	80 (1)	143		80		213	
September	94		117		100		152		147		138		156		148	
October	38		23		19		37		22		27		24		30	
Total	1357	678 (10)	1045	352 (5)	612	213 (3)	1038	285 (6)	1128	292 (4)	890	126 (2)	1035	383 (5)	1040	281 (4)

**^a^** Total rainfall (mm) at each meteorological station in each month. **^b^** Rainfall (mm) from individual rainfall events greater than 50 mm at each meteorological station in each month. ^c^ Data in parentheses indicate the number of rainstorm events.
